# Audit on Smoking Cessation in a Community Secondary Mental Health Service

**DOI:** 10.1192/bjo.2022.497

**Published:** 2022-06-20

**Authors:** Hanna Tu, Jonathan Campion

**Affiliations:** 1 South London and Maudsley NHS Foundation Trust, London, United Kingdom; 2 KU Leuven, Leuven, Belgium; 3 RCPsych Public Mental Health Implementation Centre, London, United Kingdom

## Abstract

**Aims:**

Smoking is the single largest cause of preventable death. Smoking prevalence is higher in people with mental disorders and impacts on physical health, mental health and bioavailability of psychotropic medications. Evidence-based interventions exist to support smoking cessation (SC)/reduction in people with mental disorders, although evidence suggests less provision compared to the general population. We aim to determine the unmet SC needs and associated causes in a community secondary mental health service, in order to advise appropriate service response. This audit will inform relevant work of the RCPsych Public Mental Health Implementation Centre as a case example.

**Methods:**

From the caseload of 364 patients, a sample of 91 case records was randomly selected for recording of smoking and provision of treatment. A survey of 31 smokers and 12 ex-smokers identified patient attitude and barriers in SC. Information on availability and nature of other SC provision in the community was gathered from staff and relevant services.

**Results:**

A sample of case records found 44% (n = 40) of patients were smokers compared to 13,5% in the general UK population. 31 patients were offered SC advice of whom 2 were recorded as wanting to quit. Nicotine Replacement Therapy (NRT) was offered to 13 patients and 5 were referred to SC services (SCS). Aside from smoking status, limited information on smoking was recorded.

The survey revealed that 20/31 smokers wanted to reduce or quit smoking, of whom 10 used NRT. Six were referred to SCS which helped 3 reduce. Four ex-smokers used SCS, which helped 3 to quit. Most frequently reported barriers in SC were habit, social isolation, availability of tobacco, and stress. Frequently reported enhancers in SC were NRT, allocated support with follow-up, social interventions and family support.

Regarding current service provision, we identified that local GP's did not prescribe NRT. Targeted SCS exist exclusively for inpatients and the only community SCS available offered 12 SC sessions without targeting needs of people with mental disorder.

**Conclusion:**

Despite high smoking prevalence in our caseload, there is an implementation gap in providing and recording SC advice and treatment, both in our service as in local primary care and community services. Provision of evidence-based interventions and coordination with GP's and SCS could prove useful in narrowing this gap. Results from this local project could be explored on a larger scale to address the implementation gap in SC in this population at high risk of smoking associated harm.

